# Cytoskeleton as a generator of characteristic physical properties of plant cells: ‘cell wall,’ ‘large vacuole,’ and ‘cytoplasmic streaming’

**DOI:** 10.2142/biophysico.bppb-v22.0013

**Published:** 2025-06-20

**Authors:** Amari Toshiki, Noriko Nagata, Motoki Tominaga, Hirotomo Takatsuka

**Affiliations:** 1 School of Biological Science and Technology, College of Science and Engineering, Kanazawa University, Kanazawa, Ishikawa 920-1192, Japan; 2 Department of Chemical and Biological Sciences, Faculty of Science, Japan Women’s University, Tokyo 112-8681, Japan; 3 Faculty of Education and Integrated Arts and Sciences, Waseda University, Tokyo 162-0056, Japan; 4 Graduate School of Science and Engineering, Waseda University, Tokyo 162-0056, Japan; 5 Department of Biological Sciences, Graduate School of Humanities and Sciences, Nara Women’s University, Nara 630-8506, Japan

**Keywords:** plant cell, cell wall, vacuole, cytoplasmic streaming, cytoskeleton

## Abstract

As sessile organisms, plants must constantly adapt to ever-changing environmental conditions. To survive in their habitats, plants have evolved characteristic cellular features that make the cells rigid yet dynamic. These include the cell wall, large vacuole, and cytoplasmic streaming. The cell wall is an elaborate extracellular matrix that surrounds plant cells and provides both physical strength and protection against external forces. The large vacuole is a membrane-bound organelle absent in animal cells. They can absorb water and expand, thereby exerting a force on the cell wall from within and generating turgor pressure that promotes cell expansion. In the narrow cytoplasmic space between the vacuole and the cell wall, intracellular components circulate via rapid flows, a phenomenon known as cytoplasmic streaming. In this review, we summarize how these three characteristic features of plant cells are organized with the help of cytoskeletal elements. This review article is an extended version of the Japanese article, “Cell Wall,” “Large Vacuole,” & “Cytoplasmic Streaming”: How Do Cytoskeletons Build Plant Cells with Unique Physical Properties?” by Takatsuka et al., published in SEIBUTSU BUTSURI Vol. 64, p. 132–136 (2024).

## Significance

Plant cells are mechanically stiff because of the presence of cell walls, are highly crowded due to the presence of large vacuoles, yet remain highly dynamic owing to the presence of cytoplasmic streaming. Thus, they serve as an excellent model for investigating cellular mechanics from both biophysical and physicobiological perspectives. Despite the potential, the distinctive physical properties of plant cells have received limited attention. In this review, we highlight how the plant cytoskeletons regulate the organization and behavior of cell walls, vacuoles, and cytoplasmic streaming to establish and maintain the rigid yet dynamic status of plant cells.

## Introduction

**Figure 1:**
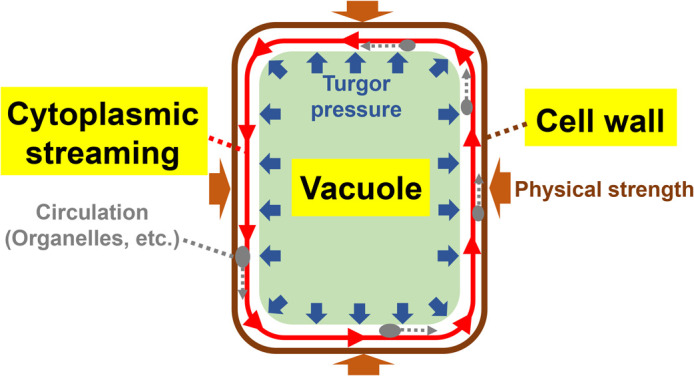
Structure of the plant cell with its characteristic physical properties. Plant cells are enclosed by rigid cell walls and harbor the large vacuole that generates large turgor pressure. Despite these structural constraints, cytoplasmic streaming is believed to play a crucial role in facilitating the circulation and distribution of cellular substances.

Unlike animals, plants are immobile and must grow and reproduce where they are rooted. Therefore, plants require sufficient mechanical strength to support the growth of stems toward the sunlight. In addition to the structural support, plants must compete with each other for resources by optimizing their structure and exhibiting high growth rates to facilitate adaptation. To achieve this, plants have evolved cell structures with specialized physical properties. Among all these structural components characteristic of plant cells, two of the most important are the plant cell wall and the large vacuole (Figure 1).

The plant cell wall comprises cellulose microfibrils, hemicellulose, pectin, lignin, and soluble proteins. Its primary role is to provide physical strength to protect them from external mechanical forces (Figure 1). Recent studies using atomic force microscopy reported that the Young’s modulus (the elastic modulus indicating the strength of an object) reached over 1 MPa in plant cells [1], compared to a maximum of ~5 kPa in animal cells [2]. These results provide quantitative evidence that plant cells are indeed more rigid than animal cells, primarily due to the presence of the cell wall.

One cellular basis for maintaining vigorous plant growth is the ability of plant cells to significantly increase in volume following the cessation of cell division (hereafter referred to as “post-mitotic cell growth” or more simply “cell growth”). In some cases, individual plant cells can increase their volume by 1,000-fold or more, with specific cell types reaching lengths over 1 mm. This is in stark contrast to animal cells, most of which do not significantly change in size following cell division. The driving force behind plant cell growth is turgor pressure, the internal force exerted by water that pushes the plasma membrane against the cell wall. Turgor pressure in plant cells can be extremely high, reaching up to 5 MPa in some cells; far exceeding the air pressure of typical automobile tires (~0.2 MPa) [3–6]. A major source of turgor pressure is the large vacuole, a membrane-bound organelle, functionally specialized in the plant cell. Vacuoles, which are absent in animal cells, can increase in size through repeated fusion events and water uptake, eventually occupying more than 90% of the total cell volume.

However, large vacuoles can also pose significant challenges to plants. As the vacuole expands, the cytoplasm, which accounts for <10% of the total cell volume, is pushed to the periphery. This localization physically restricts the movement of substances through the center, especially in differentiated plant cells. To overcome this challenge, plant cells employ cytoplasmic streaming, an active and directed flow of the cytoplasm around the large vacuole, which promotes the efficient intracellular circulation. This view is supported by the observation that cytoplasmic streaming correlates with cell size, being prominent in large, vacuolated cells, but rarely seen in smaller, cytoplasm-rich cells [7]. The classic example is *Chara braunii*, where internodal cells can reach up to 10 cm long and exhibit rapid (100 μm/s) flow along the cell periphery [7]. In contrast, cytoplasmic streaming is rare in animal cells, limited to only in a small fraction of cell types, such as the oocytes of *Drosophila melanogaster* and *Caenorhabditis elegans* [7]. This indicates cytoplasmic streaming as a distinct physical feature of plant cells.

This review summarizes the current knowledge on how cytoskeletal elements regulate the organization and dynamics of the cell wall, large vacuoles, and cytoplasmic streaming. We also explore the key unsolved questions and emerging studies related to these characteristic plant cell features.

## Cytoskeletons in plants

The cytoskeleton is a dynamic network of filaments or fibers located in the cell. It plays crucial roles in eukaryotic cells, including maintaining cell shape and facilitating the intracellular transport of vesicles and organelles, as well as various cellular functions. The cytoskeleton consists of three major filament systems: actin filaments (AFs), microtubules (MTs), and intermediate filaments (IFs) [8,9]. AFs, also known as microfilaments, are the thinnest cytoskeletal elements with a diameter of approximately 7 nm. They comprise G (globular)-actin subunits that polymerize into a pair of intertwined strands [8]. Myosin, a family of mechano-enzymatic motor proteins, hydrolyzes adenosine triphosphate (ATP) to generate force and movement, allowing them to move along AFs [8]. MTs are the thickest cytoskeletal filaments approximately 25 nm in diameter. They comprise α- and β-tubulin heterodimers that polymerize to form hollow cylindrical filaments. In animal cells, the motor proteins dynein and kinesin hydrolyze ATP to transport cargo along MTs [8]. Most kinesins move toward the plus end of MTs, whereas dyneins are “minus-end directed motors.” Interestingly, plants have lost dynein genes [10]. Instead, the minus-end-directed transport in plants is mediated by members of the plant-specific class VI kinesin-14 family [11]. Finally, IFs are named for their intermediate thickness (~10 nm) between AFs and MTs. To date, no canonical IFs or their equivalents have been identified in plants [9]. However, a recent study reported an *Arabidopsis* gene encoding a protein with the motifs characteristic of the IFs is capable of forming cytoskeleton-like filamentous structures [12]. Whether IFs exist in plants remains an open question. Overall, the structures of plant G-actin and tubulin proteins and thus AFs and MTs are highly similar to those of animal cells [13,14]. Nonetheless, plant cytoskeletons have acquired unique functions during evolution, such as supporting cell wall formation, regulating the structures of large vacuoles, and driving cytoplasmic streaming.

## How cytoskeletons help to build elaborate cell wall structures

Upon completion of cell division, the plant cells undergo differentiation typically accompanied by turgor pressure-driven irreversible cell growth. During this phase, plant cells often undergo dramatic changes in their shape, which are closely linked to with their specialized function. A classic example is the jigsaw-puzzle-like appearance of mature leaf epidermal cells, which are frequently observed in dicot plants. This unique shape results from differential growth of initially undifferentiated polygonal cells. In these cells, specific sites actively grow to form outward protrusions or lobes while others grow slowly, creating indentations [15,16]. The formation of lobes and indentations leads to an interlocking pattern among neighboring cells, which is believed to improve the tissue’s ability to confer physical resistance, particularly to the pulling forces experienced in the leaf tissues.

**Figure 2:**
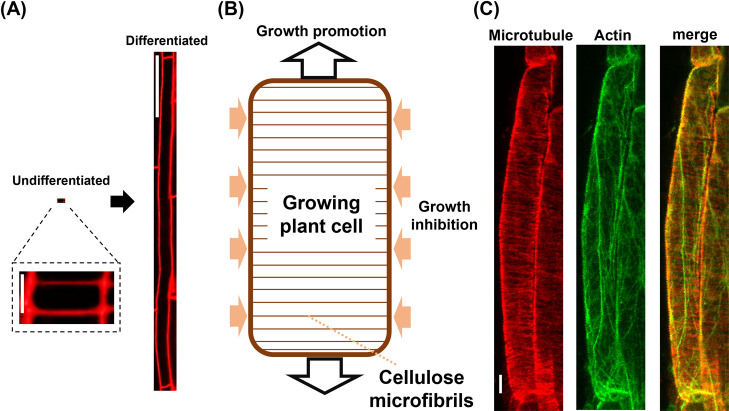
Organization of the cell walls and cytoskeletons in elongating root cells. (A) An undifferentiated cell just after division (left) and a fully differentiated cell (right) from the root epidermis of *Arabidopsis thaliana*. Cell outlines were visualized using propidium iodide. Scale bars represent 5 μm (left) and 100 μm (right). (B) In root cells elongating longitudinally, cellulose microfibrils align transversely at the cell cortex, restricting lateral expansion and supporting directional growth. (C) Immunofluorescence images of a growing root cell of *Arabidopsis thaliana* simultaneously stained with anti-tubulin and anti-actin antibodies. MTs are oriented transversely (left), while AFs are oriented longitudinally (middle). The merged image is shown on the right. Scale bar: 10 μm.

The cell wall not only provides mechanical strength but also plays a central role in determining the direction of cell growth. It primarily comprises cellulose microfibrils, linear polymers made of thousands of D-glucose units connected via β-1,4 glycosidic bonds [17]. Within plant cells, these cellulose microfibrils function like the metal hoops that tighten a barrel; in essence, they restrict cell growth against the direction of their orientation [18]. For example, in undifferentiated, square-shaped, small root cells, cellulose microfibrils are oriented transversely prior to the onset of cell growth. This orientation resists lateral cell expansion and promotes longitudinal growth, facilitating the production of highly elongated and thin differentiated cells (Fig. 2A, B) [18].

MTs play a crucial role in determining the orientation of cellulose microfibrils. These microfibrils are synthesized by plasma membrane-localized cellulose synthase complexes (CSCs), which track along the cortical MTs during cellulose synthesis [19]. Hence, the MTs act as scaffolds to guide CSCs’ movement, thus directing the orientation of newly synthesized cellulose microfibrils [19]. By doing so, MTs determine the cell shape by preventing the expansion in a direction that may lead to improper growth. Consistent with this, inhibition of MT polymerization by the drug oryzalin causes abnormally swollen root cells, which generally grow in a long and thin shape [20].

In contrast to the inhibitory role MTs play in cell growth, actin plays a promotive role by facilitating the transport of materials required for cell growth [21,22]. Previous studies showed that the stepwise cytoskeletal changes drive the elongation of root cells along the apical-basal axis [22,23]. First, MTs aligned along the transverse direction to restrict swelling, followed by the longitudinal alignment of AFs to facilitate elongation [22]. These mutually perpendicular MTs and AFs in elongating root cells can be visualized using a dual immunostaining technique developed recently (Figure 2C) [24]. Collectively, there is considerable evidence that coordinated action of AFs and MTs determines the diversity of plant cell shapes.

Given their crucial role in morphogenesis, MTs undergo regulated and rapid reorganization in response to internal and external cues. For instance, treatment with the plant hormone auxin, which inhibits longitudinal cell growth in roots, leads to a massive reorientation of MTs from transverse to the longitudinal direction. This process begins within 10 min of treatment and is completed within an hour, suggesting that it may be accomplished via a nontranscriptional process [25]. A recent study identified small GTPase signaling as a molecular basis for the short-term 90° MT reorientation [26]. Specifically, RHO-OF-PLANTS6 (ROP6), a member of the plant-specific small GTPases, along with its effector ROP-interactive CRIB motif-containing protein 1 (RIC1), promotes MT reorganization by activating the MT-severing protein katanin [26]. Despite decades of research, the precise molecular mechanisms underlying the rapid MT reorganization remain poorly understood. Uncovering these mechanisms may lead to novel technologies for manipulating plant cell shape with high precision.

## How cytoskeletons regulate the morphological changes of large vacuoles

**Figure 3:**
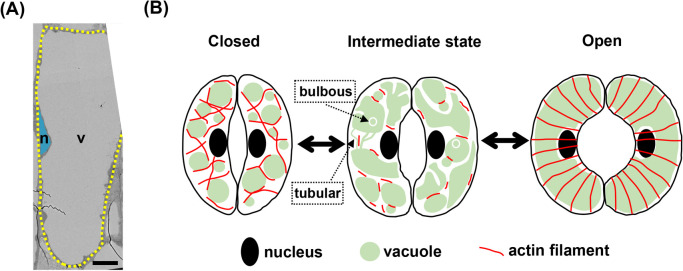
Large vacuoles in plant cells and their dynamics during stomal opening and closing. (A) A ransmission electron micrograph (TEM) of a root epidermal cell of *Arabidopsis thaliana*. The cell outline was traced with dotted yellow lines. The scale bar represents 10 μm. n: nucleus (shown in blue), v: vacuole (shown in white). (B) Dynamics of AFs and vacuoles during stomatal opening and closing.

As mentioned above, the plant vacuole occupies a significant portion of the total cell volume and exerts an internal pressure, thereby contributing to cell growth (Figure 3A). In addition to its fundamental roles in storing various substances and sequestering waste products [27], the vacuole in plant cells also plays a significant role in regulating cell size and growth, making it an essential organelle in plant cells. Stomata, small pores on the leaf surface that facilitate gas exchange, serve as a valuable model for understanding vacuolar dynamics. Upon sensing environmental cues, a pair of kidney-shaped guard cells that form the stoma undergo swelling and shrinking, resulting in stomatal opening and closing, respectively [28]. Stomatal opening is primarily driven by proton pump activation, ion influx, and osmotic changes. In addition, dynamic structural changes in the vacuoles within each guard cell also drive stomatal movement. During stomatal opening, multiple small vacuoles fuse into a large vacuole, thereby generating a turgor pressure as high as 5 MPa to open the stomata (Figure 3B) [29]. Conversely, during closure, the large vacuole can split into many small, globular vacuoles (Figure 3B) [29]. Transitional vacuolar structures, such as tubular and bulbous vacuoles, are also observed during the intermediate states between open and closed stomata (Figure 3B) [29].

It is noteworthy that such vacuolar transformation takes place in as little as 30 min during the stomatal movement. Such rapid structural changes have been reported to be actin-dependent. As shown in Figure 3B, the AFs are randomly oriented in the closed stomata, while they exhibit a radial pattern in the open stomata [29]. During the transition between these states, AFs undergo temporary disassembly [29]. In line with this, treatment with actin-stabilizing drug delays both stomatal opening and closure, suggesting that temporary disassembly of AFs is a crucial step in rapid stomatal movement [29].

How does actin alter vacuolar structures? Careful observations by Wang *et al.* showed that AFs surround individual small, globular vacuoles and disassemble during their fusion [30], suggesting that AFs may form barriers to vacuolar fusion. Interestingly, actin rings are also formed at the contact sites between the actively fusing vacuoles, implying that actin also promotes vacuolar fusion [30]. Similar dual functions of AFs in vacuolar dynamics have also been proposed in other types of plant cells. However, the mechanisms by which AFs exert their opposing functions remain completely unknown.

From a biophysical perspective, it is important to determine how actin generates the force required to deform the vacuoles that are actively absorbing water and increasing in volume. Vacuoles are known to store enzymes that possess caspase-like activity and can therefore induce cell death if these enzymes are released into the cytosol [31]. Therefore, during rapid vacuolar remodeling, AFs must exert a force that exceeds the internal vacuolar pressure while preserving the integrity of the tonoplasts. Further research focusing on the actin-based mechanisms that precisely control vacuole dynamics is crucial for a better understanding of the unique biophysical environments inside plant cells.

## How cytoskeletons support cytoplasmic streaming

In large, differentiated plant cells, the cytosol is confined to the thin layer between the cell wall and the large vacuole (Figure 3A). In such a constrained environment, passive diffusion alone is insufficient for the efficient circulation of cellular nutrients, organelles, and other substances, nor can it support rapid cellular responses. Hence, active transport is required, making cytoplasmic streaming crucial. Cytoplasmic streaming refers to an active, directional flow of cytoplasm observed across a broad group of organisms, ranging from algae to angiosperms [7]. Generally, the rate of cytoplasmic streaming increases with cell size, presumably to ensure adequate intracellular circulation [7,32]. In angiosperms, for example, organelles such as mitochondria and Golgi bodies move at a rate of approximately 1–10 μm/s due to cytoplasmic streaming [33,34]. Remarkably, when scaled up to human dimensions, the speed of organelle movement through the highly crowded, narrow cytoplasmic spaces is comparable to that of the fastest human sprinter (about 10 m/s).

**Figure 4:**
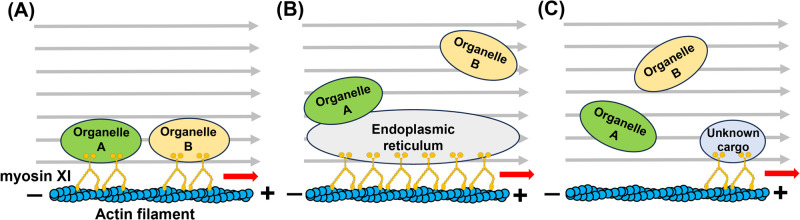
Hypothetical models of actomyosin-driven cytoplasmic streaming. Red arrows indicate the direction of myosin movement along AFs, while grey arrows represent the resulting cytoplasmic flow.

Previous research has demonstrated that specific actomyosin-based machinery drives cytoplasmic streaming within plant cells [35]. Plant myosins are classified into two groups that are uniquely found in plants: class XI and class VIII. Among these, myosin XI has been implicated in cytoplasmic streaming [35]. Plant myosin XI moves processively along the AFs, taking 35-nm steps at a velocity of approximately 7 μm/s [36]. This movement is equivalent to a human running at a speed of 200 m/s with 1 m stride. The current model of cytoplasmic streaming is as follows: (1) Myosin XI binds to a target organelle and moves toward the plus end of AFs, powered by ATP hydrolysis. Next, (2) the movement of organelles in the viscous cytoplasm, driven by myosin, generates a flow in the surrounding cytoplasm. Together, these events generate a directed cytoplasmic flow, i.e., cytoplasmic streaming [37,38]. Despite its functional significance, the exact mechanism by which the myosin carries target organelles and generates cytoplasmic streaming remains to be elucidated, partly due to the technical difficulty in visualizing myosin molecules *in vivo*. To date, three main hypotheses have been proposed: (1) Myosin XI directly binds to various organelles, including mitochondria, peroxisomes, and chloroplasts, and transports them directly along AFs (Figure 4A); (2) Myosin XI moves the endoplasmic reticulum (ER), which has large surface area; consequently, organelles physically interacting with the ER are co-transported (Organelle A in Figure 4B). In addition, ER movement induces cytoplasmic flow, which allows the movement of other organelles without directly interacting with the ER (Organelle B in Figure 4B). Finally, (3) Myosin XI transports an unknown cargo to generate a cytoplasmic flow, thereby facilitating the movement of other organelles (Figure 4C) [39,40]. Based on the observations, this unknown cargo is likely larger than a vesicle but smaller than mitochondria or chloroplasts [41,42]. These hypotheses are not mutually exclusive, and the relative contribution of each mechanism may vary across different plant species and cell types.

Cytoplasmic streaming is unique compared to other intracellular transport mechanisms, leading to extensive investigations into how it is initiated. However, comparatively less attention has been paid to understanding its physiological roles in plants [43]. A recent study has revealed the crucial role of cytoplasmic streaming in determining plant biomass production. The expression of chimeric myosin XIs, constructed by substituting the motor domain of *Arabidopsis thaliana* myosin XI-2 with that of *Chara corallina* myosin XI (a fast myosin) or *Homo sapiens* myosin V (a slow myosin) in Arabidopsis *myosin xi-2* mutants, led to faster and slower cytoplasmic streaming, respectively, relative to wild-type myosin XI-2 cells [44,45]. Importantly, the velocity of cytoplasmic streaming is positively correlated with plant size. Specifically, transgenic plants exhibiting faster cytoplasmic streaming show enhanced cell growth, leading to a larger plant size. In line with this, plants with slower cytoplasmic streaming result in reduced cell size and consequently smaller plant bodies [44,45]. Cytoplasmic streaming is believed to facilitate efficient intracellular distribution of substances essential for cell expansion, including nutrients, metabolites, cell-wall precursors, and plant hormones, thereby influencing cell size [44,45].

In the future, biophysical research integrating advanced imaging techniques and mathematical modeling approaches may help uncover the molecular mechanisms behind cytoplasmic streaming and characterize its physiological roles.

## Conclusions

This review has explored the current research on several distinct physical properties of plant cells, including cell walls, large vacuoles, and cytoplasmic streaming. While plant cells are structurally stabilized by their cell walls and are highly crowded due to large vacuoles, they are remarkably dynamic, primarily due to cytoplasmic streaming. Among various cellular processes, post-mitotic cell growth requires precise coordination among cell walls, large vacuoles, and cytoplasmic streaming. This process drives the vigorous growth of plants, such as bamboo that grow more than one meter each day [46–48], and is precisely regulated in response to environmental cues [49,50]. Therefore, understanding this coordination from a biophysical perspective is not only of fundamental scientific interest but also crucial for applied research aimed at sustainable crop production. The extensibility of cell walls can be optimized to accommodate expanding vacuoles, allowing cells to enlarge. Meanwhile, cytoplasmic streaming spontaneously occurs as plant cell size increases, further supporting cell growth. Based on the evidence reviewed here, the cytoskeletons most likely play a central role in orchestrating the interplay between cell wall patterning, vacuolar morphology, and cytoplasmic streaming dynamics during plant cell growth.

Overall, the characteristic physical features of plant cells deserve more attention as a subject of biophysical research. Differentiated plant cells, characterized by limited cytoplasmic space constrained between rigid cell walls and large vacuoles, provide an excellent model for assessing how crowded cellular environments, common among prokaryotic and eukaryotic cells, are resolved. Future studies focusing on plant cell architecture and dynamics may offer novel insights into their nature from biophysical and physicobiological perspectives.

## Conflict of Interest

The authors declare that there are no conflicts of interest.

## Author Contributions

T.A., N.N., M.T., and H.T. wrote the manuscript and prepared figures.

## Data Availability

The evidence data generated and/or analyzed during the current study are available from the corresponding author on reasonable request.

## Acknowledgements

This work was supported by MEXT KAKENHI (grant number 22K06293), and JST PRESTO (grant number JPMJPR22E6) to H.T. and WISE Program for Nano-Precision Medicine, Science, and Technology of Kanazawa University by MEXT to T.A.
